# GANT61 Modulates Autophagy and Lipid Metabolism in Ovarian Cancer

**DOI:** 10.1111/cpr.70051

**Published:** 2025-05-01

**Authors:** Yibin Pan, Lingfeng Chen, Jinlu Shen, Shihao Hong, Xiaojing Guan, Xudong Ma, Rongrong Tang, Meifei Lu, Fangying Sun, Shanliang Shang, Yongdong Dai, Zhaokai Zhou, Songying Zhang, Jianhua Yang

**Affiliations:** ^1^ Assisted Reproduction Unit, Department of Obstetrics and Gynecology, Sir Run Run Shaw Hospital, School of Medicine Zhejiang University Hangzhou People's Republic of China; ^2^ Zhejiang Provincial Clinical Research Center for Obstetrics and Gynecology Hangzhou People's Republic of China; ^3^ Zhejiang Key Laboratory of Precise Protection and Promotion of Fertility Hangzhou People's Republic of China; ^4^ Department of Obstetrics and Gynecology Wenling First People's Hospital Wenling People's Republic of China; ^5^ Department of Pharmacy, the Children's Hospital Zhejiang University School of Medicine Hangzhou People's Republic of China; ^6^ Department of Urology The Second Xiangya Hospital of Central South University Changsha Hunan People's Republic of China

## Abstract

GANT61 induced autophagy via the AKT pathway and promoted the accumulation of lipid droplets in both cell lines. The molecular mechanism behind this lipid accumulation appears to involve the mediation of SREBP1. Furthermore, the combination of GANT61 with CQ/Fatostatin significantly inhibited the proliferation and clonogenicity of SKOV3 and SKOV3PTX cells.

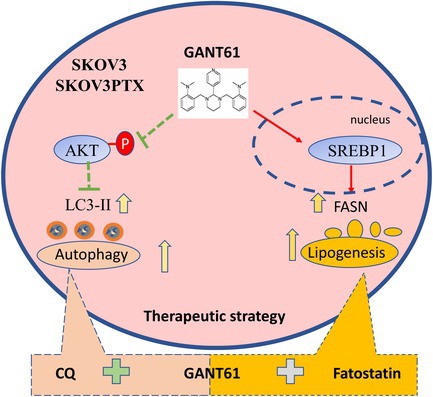

**Abbreviations**
c‐SREBP1: cleaved SREBP1
EOC: epithelial ovarian cancer
FASN: fatty acid synthase
Hh: Hedgehog
LDs: lipid droplets
OC: ovarian cancer
PTX: paclitaxel
RTCA: real‐time cell analysis
RT‐qPCR: reverse transcription quantitative polymerase chain reaction
SHH: sonic Hedgehog
siRNA: small interfering RNA
SREBP1: sterol regulatory element‐binding protein 1
TEM: transmission electron microscopy
WB: Western blot

Dear editor,

Ovarian cancer (OC) remains one of the leading causes of mortality among women with gynaecological malignancies worldwide. Its high lethality stems from its insidious onset, lack of early biomarkers and asymptomatic early stage progression [1, 2, 3]. Among the various histopathological subtypes, epithelial ovarian cancer (EOC) accounts for 85%–90% of cases, with high‐grade serous ovarian carcinoma comprising approximately 75% of all EOC cases [4]. Despite progress in targeted therapies, such as PARP inhibitors for BRCA‐mutated cases, anti‐angiogenic agents and folate receptor‐targeted antibody‐drug conjugates, platinum‐taxane chemotherapy with cytoreductive surgery remains the cornerstone of treatment [5, 6]. However, clinical outcomes in OC remain suboptimal, with up to 20% of patients exhibiting resistance during initial treatment and 50%–80% experiencing disease relapse, often within 2 years [7]. This relapse significantly reduces the 5‐year survival rate of advanced OC patients to a dismal 30%–40% [8].

Emerging evidence suggests that interactions between the Hedgehog pathway, lipid metabolism, and autophagy contribute to some cancer progression [9, 10, 11, 12, 13, 14, 15, 16]. However, the precise mechanisms remain poorly understood. This study aims to explore the combined effects of GANT61 and chloroquine (CQ) or Fatostatin on OC cells, providing potential therapeutic strategies for advanced OC.

To address this question, we first determined the IC50 value of GANT61 (a GLI1‐induced transcriptional inhibitor that suppresses the Hedgehog signalling pathway) in SKOV3 and SKOV3PTX using the CCK‐8 assay. The IC50 value of GANT61 was 17 μM for SKOV3, while it increased to 23 μM in SKOV3PTX (Figure 1A). Real‐time cell analysis (RTCA) revealed that GANT61 inhibited cell proliferation in a dose‐dependent manner (Figure 1B). We further confirmed that GANT61 significantly suppressed GLI1 expression at both the mRNA and protein levels in both cell lines (Figure 1C,D). These results support the idea that GANT61 exerts its inhibitory effects by targeting the Hedgehog pathway and reducing GLI1‐mediated transcription in both cell lines. Colony formation assays further revealed that GANT61 suppressed the clonogenic potential of SKOV3 and SKOV3PTX cells (Figure 1E). Notably, Western blot (WB) analysis revealed increased SHH (Sonic Hedgehog)–N and GLI1 proteins in SKOV3PTX cells compared to SKOV3 cells (Figure 1F), suggesting compensatory Hh pathway action as a potential resistance mechanism to paclitaxel. Next, we investigated whether GANT61 can induce autophagy in SKOV3 and SKOV3PTX cells. Transmission electron microscopy (TEM), the gold standard for autophagy assessment, revealed a significant increase in double‐membrane autophagic vacuoles in GANT61‐treated SKOV3 and SKOV3PTX cells versus controls, containing intact cytoplasmic material (Figure 2A). This autophagosome accumulation was further confirmed by immunoblotting showing LC3B‐II (autophagosome marker) conversion (Figure 2B). We further observed a dose‐ and time‐dependent increase in P62 and LC3B‐II protein levels upon GANT61 treatment, with CQ cotreatment further elevating P62 and LC3B‐II levels (Figure 2C) and enhancing colocalization of P62 and LC3B‐positive autophagosomes in both SKOV3 and SKOV3PTX cells by immunofluorescence staining (Figure 2D). The mCherry‐GFP‐LC3B dual‐fluorescence reporter (where autophagosomes exhibit both GFP+/mCherry+ signals and autolysosomes retain only mCherry+ due to GFP quenching in acidic compartments) confirmed these findings, showing that GANT61 treatment increased yellow puncta (autophagosomes), with CQ cotreatment further elevating yellow puncta (Figure 2E). These data collectively demonstrate that GANT61 simultaneously promotes autophagic flux initiation while inhibiting lysosomal acidification. Mechanistically, GANT61 treatment elicited a dose‐ and time‐dependent suppression of AKT activation, as evidenced by reduced phosphorylation at both Ser473 and Thr308 in SKOV3 cells, with similar but attenuated effects observed in SKOV3PTX cells (Figure 2F). Maximal inhibition was achieved at 10 μM after 24 h treatment (Figure 2F). This dual‐site dephosphorylation pattern suggests comprehensive disruption of AKT membrane translocation and kinase activation.

**FIGURE 1:**
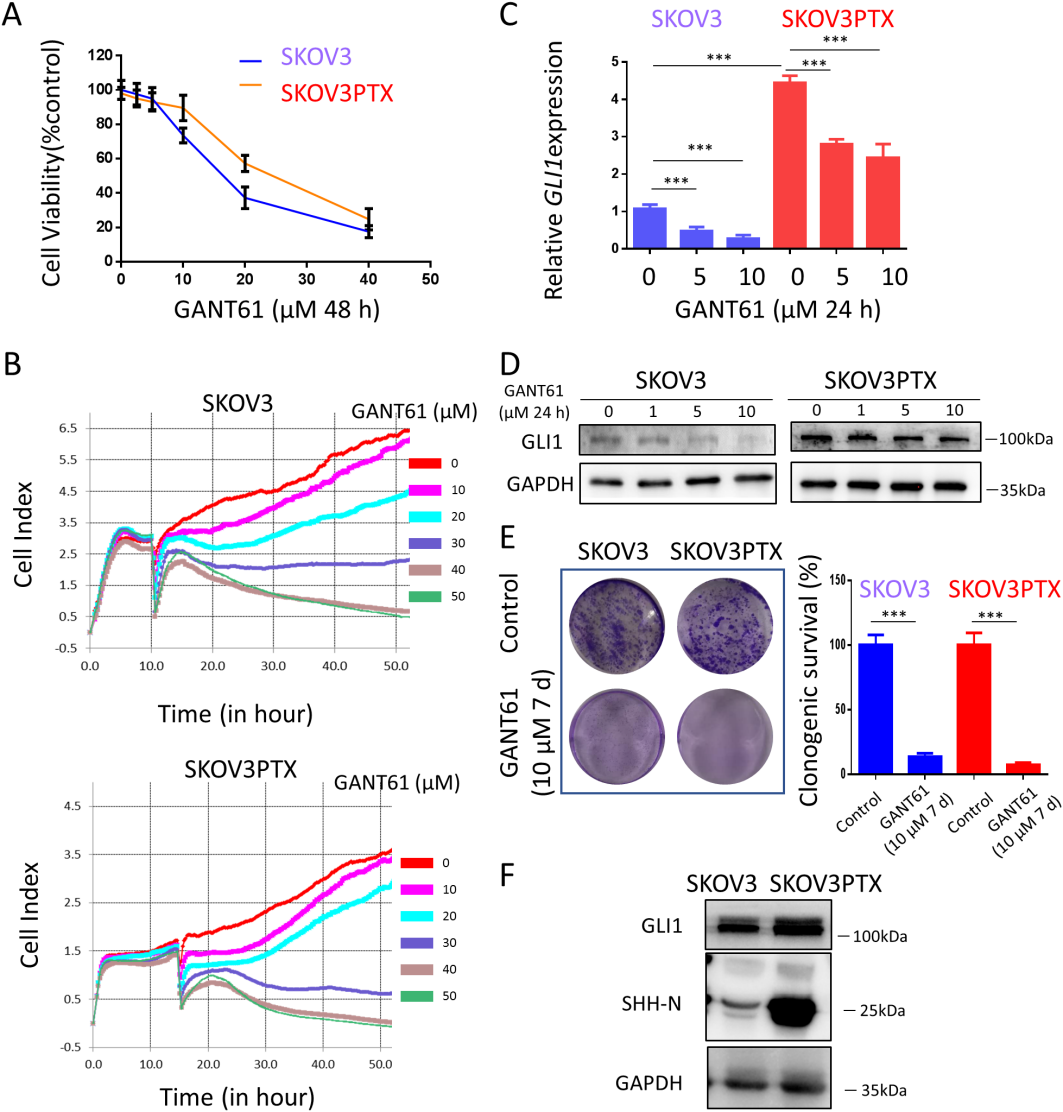
GANT61 inhibits proliferation and colony formation in SKOV3 and SKOV3PTX cells. (A) SKOV3 and SKOV3PTX cells were treated with GANT61 at concentrations of 0, 1, 5, 10, 20, 30 and 40 μM for 48 h. Cell viability was expressed as a percentage relative to untreated control cells (set as 100%) and IC50 values were calculated based on the 48‐h treatment. (B) SKOV3 and SKOV3PTX cells were treated with GANT61 (0, 10, 20, 30, 40 and 50 μM), and cell proliferation curves were monitored in real‐time using RTCA. (C) SKOV3 and SKOV3PTX cells were treated with GANT61 (0, 5 and 10 μM) for 24 h, and *GLI1* mRNA levels were measured using RT‐qPCR. Data are presented as the mean ± SD of three independent experiments, based on two‐way factorial ANOVA. (D) SKOV3 and SKOV3PTX cells were treated with GANT61 (0, 1, 5 and 10 μM) for 24 h, and GLI1 protein levels were assessed by WB, with GAPDH as a loading control. (E) SKOV3 and SKOV3PTX cells were treated with 10 μM GANT61 for 7 days, and colony formation assays were performed to evaluate the impact on colony‐forming ability. (F) The expression levels of GLI1 and SHH proteins in SKOV3 and SKOV3PTX cells were analysed by WB to compare the Hedgehog pathway activity in the two cell lines. *p < 0.05, **p < 0.01, ***p < 0.001.

**FIGURE 2:**
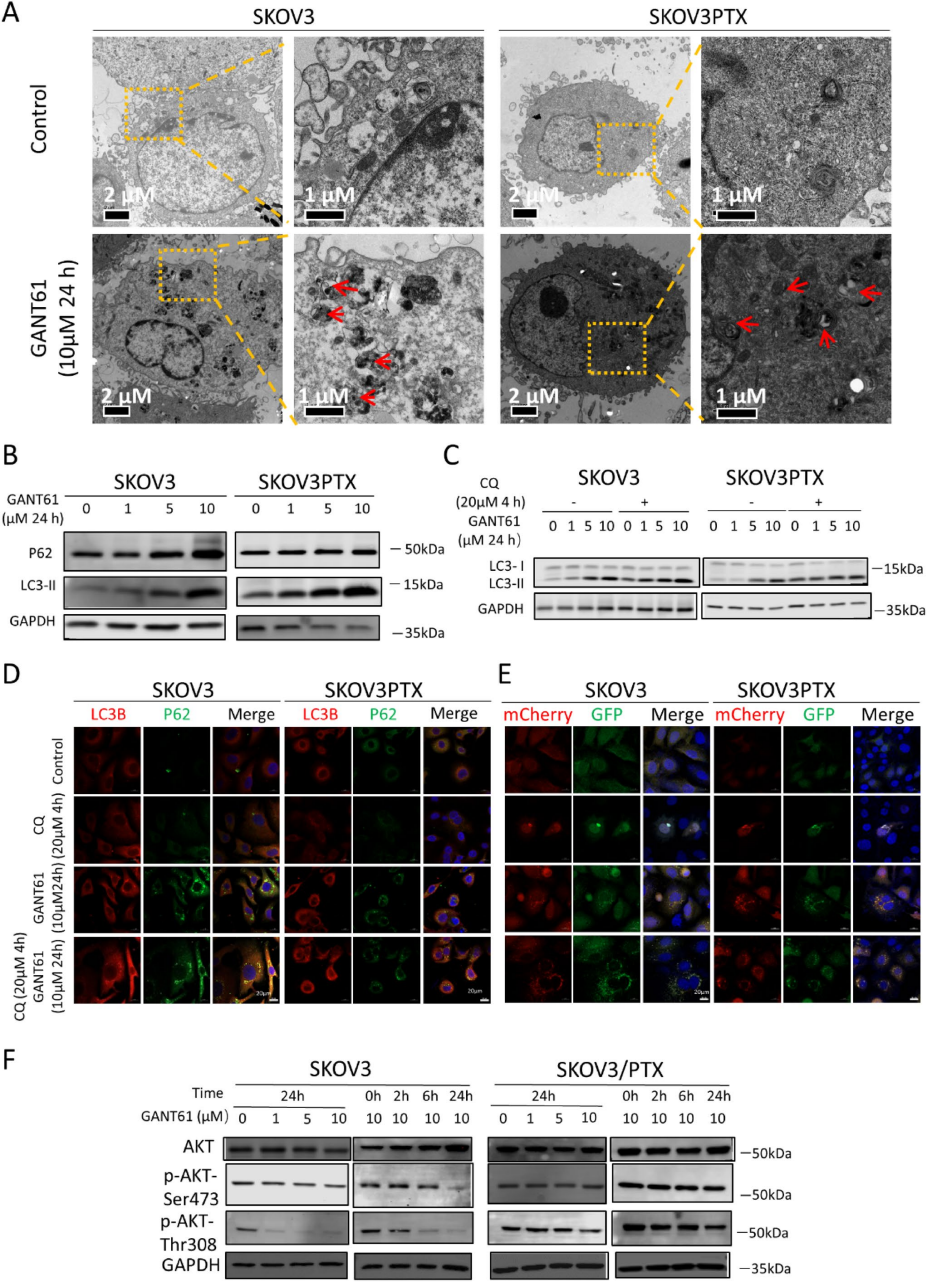
GANT61 promotes autophagosome formation in SKOV3 and SKOV3PTX cells by inhibiting the AKT signalling pathway. (A) TEM images of SKOV3 and SKOV3PTX cells treated with 10 μM GANT61 for 24 h. Representative double‐membrane autophagosomes are observed, scale bar 1 μm, scale bar =2 μm. (B) WB analysis of P62 and LC3B‐II protein levels in SKOV3 and SKOV3PTX cells treated with GANT61 (0, 1, 5 and 10 μM) for 24 h. GAPDH was used as a loading control. (C) WB detection of LC3B‐II levels in SKOV3 and SKOV3PTX cells treated with GANT61 (0, 1, 5 and 10 μM) for 24 h, with or without CQ (20 μM). For combined treatment, CQ was added 20 h after GANT61 treatment and incubated for an additional 4 h. GAPDH was used as a loading control. CQ, chloroquine, an autophagy inhibitor. (D) Immunofluorescence analysis of LC3B (red) and P62 (green) in SKOV3 and SKOV3PTX cells under different treatment conditions: Control, CQ (20 μM for 4 h), GANT61 (10 μM for 24 h), and GANT61 combined with CQ (10 μM GANT61 for 24 h + 20 μM CQ for the final 4 h). Hoechst (blue) was used to stain nuclei. Scale bar = 20 μm. (E) Confocal microscopy of autophagic flux using mCherry‐GFP‐LC3B dual fluorescence reporter in SKOV3 and SKOV3PTX cells after 24 h of infection. Cells were divided into four groups: Control, CQ (20 μM for 4 h), GANT61 (10 μM for 24 h) and GANT61 combined with CQ (10 μM GANT61 for 24 h + 20 μM CQ for the final 4 h). Hoechst (blue) was used to stain nuclei. Scale bar = 20 μm. (F) WB analysis was performed to detect the protein levels of AKT, p‐AKT (Ser473) and p‐AKT (Thr308), with GAPDH used as a loading control. SKOV3 and SKOV3PTX cells were treated with 0, 1, 5 and 10 μM GANT61 for 24 h. Additionally, cells were treated with 10 μM GANT61 for 0, 2, 6 and 24 h. *p < 0.05, **p < 0.01, ***p < 0.001.

To explore the role of autophagy in GANT61‐mediated cytotoxicity, we blocked autophagosome‐lysosome fusion by CQ. RTCA results showed that combining GANT61 with CQ significantly reduced cell viability in both SKOV3 and SKOV3PTX cells compared to the GANT61 treatment alone (Figure 3A). CQ alone had no significant effect on proliferation, suggesting that autophagy inhibition specifically sensitises cells to GANT61 treatment (Figure 3A). Immunofluorescence staining showed that GANT61 treatment increased P62 or LC3B colocalization with LAMP1 (lysosomal marker), confirming autolysosome formation (Figure 3B,C). Cotreatment with CQ further augmented lysosomal aggregation, suggesting impaired autophagic degradation. These findings suggest that combined Hedgehog pathway inhibition and autophagy targeting may represent a promising therapeutic strategy for OC.

**FIGURE 3:**
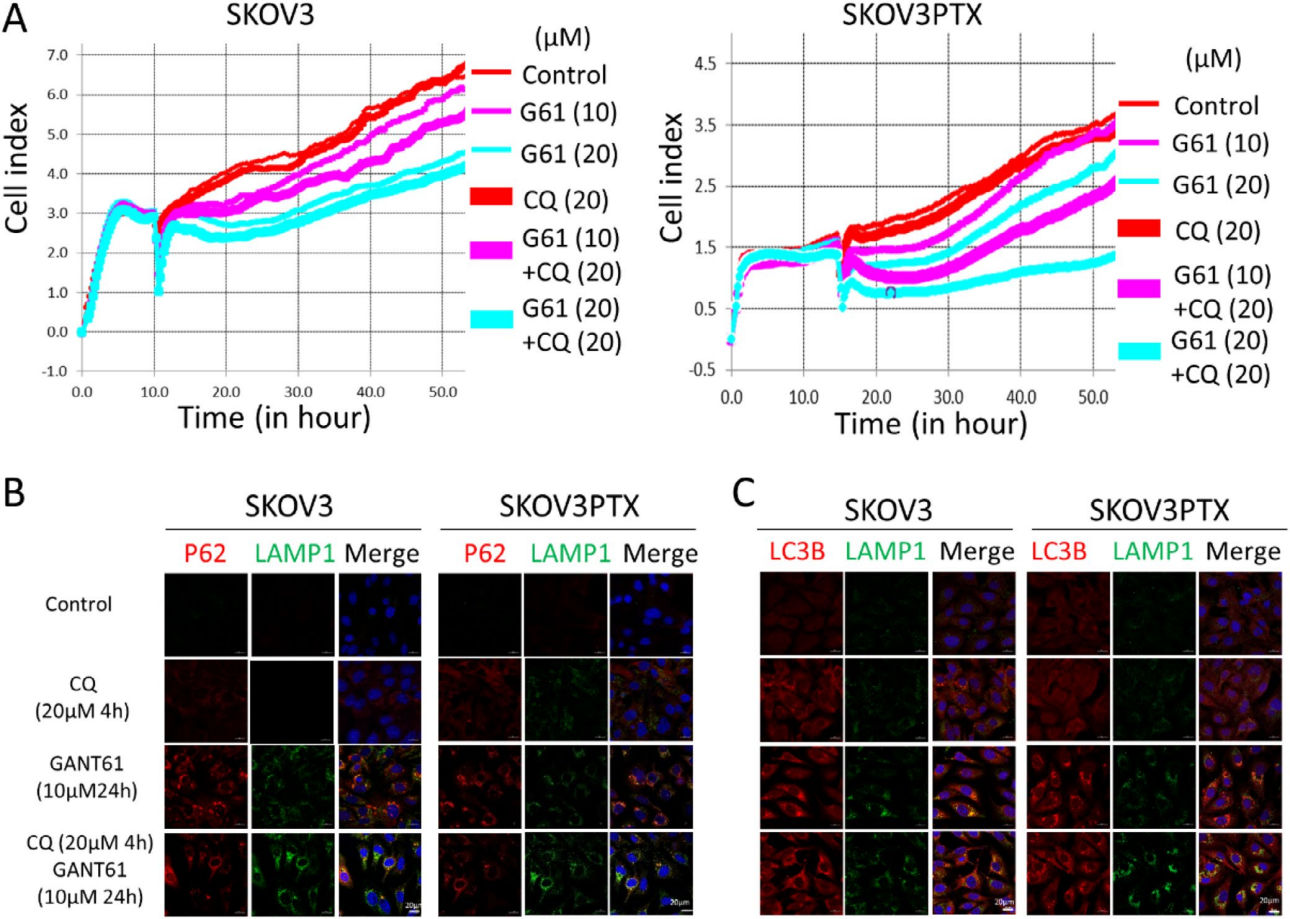
Inhibition of autophagy enhances GANT61‐induced reduction in cell viability of SKOV3 and SKOV3PTX cells. (A) Real‐time cell analysis (RTCA) of cell proliferation curves under various treatments: Control, CQ (20 μM), GANT61 (10 μM), GANT61 (10 μM) + CQ (20 μM), GANT61 (20 μM) and GANT61 (20 μM) + CQ (20 μM). (B) Confocal microscopy of immunofluorescence staining for P62 (red) and LAMP1 (green) in SKOV3 and SKOV3PTX cells under different conditions: Control, CQ (20 μM for 4 h), GANT61 (10 μM for 24 h) and GANT61 (10 μM) + CQ (20 μM for the final 4 h). Hoechst (blue) was used to stain nuclei. Scale bar = 20 μm. (C) Confocal microscopy of immunofluorescence staining for LC3B (red) and LAMP1 (green) in SKOV3 and SKOV3PTX cells treated with control, CQ (20 μM for 4 h), GANT61 (10 μM for 24 h), and GANT61 (10 μM) + CQ (20 μM for the final 4 h). Hoechst (blue) was used to stain nuclei. Scale bar = 20 μm.

Dysregulated lipogenesis, driven by fatty acid synthase (FASN) overexpression, promotes tumour progression and is associated with poor clinical outcomes across multiple cancer types, including ovarian and other malignancies [17, 18, 19, 20, 21, 22]. Thus, we examined the relationship between the Hedgehog pathway and lipid metabolism. GANT61 treatment markedly increased both mRNA and protein expression of FASN in SKOV3 and SKOV3PTX cells, indicating its role in modulating lipid metabolism (Figure 4A,B). Lipid droplets (LDs) accumulation induced by GANT61 treatment was visualised using BODIPY and Nile Red staining and confirmed by TEM (Figure 4C,D). RT‐qPCR analysis revealed upregulation of SREBP1 target genes (ACLY, LDLR, FASN, HMGCR, SCD1, GPAM and INSIG), indicating Hedgehog signalling can regulate lipid metabolism (Figure 4E). These effects were reversed by Fatostatin, a pharmacological SREBP1 inhibitor, indicating the involvement of SREBPs in GANT61‐induced lipogenesis (Figure 4E). Immunohistochemical analysis revealed significantly higher FASN expression in OC tissues compared to benign lesions and normal ovarian epithelium (Figure 4F), suggesting FASN as both a metabolic vulnerability and potential prognostic biomarker in OC. To further explore the role of SREBP1 in GANT61‐induced LDs accumulation, we performed siRNA‐mediated knockdown of SREBP1 in SKOV3 cells (confirmed by WB, Figure 4G). Both BODIPY and Nile Red staining revealed SREBP1 knockdown abrogated GANT61‐induced LDs accumulation in SKOV3 cells (Figure 4H,I), indicating that GANT61‐induced LDs formation is via SREBP1‐dependent lipogenesis.

**FIGURE 4:**
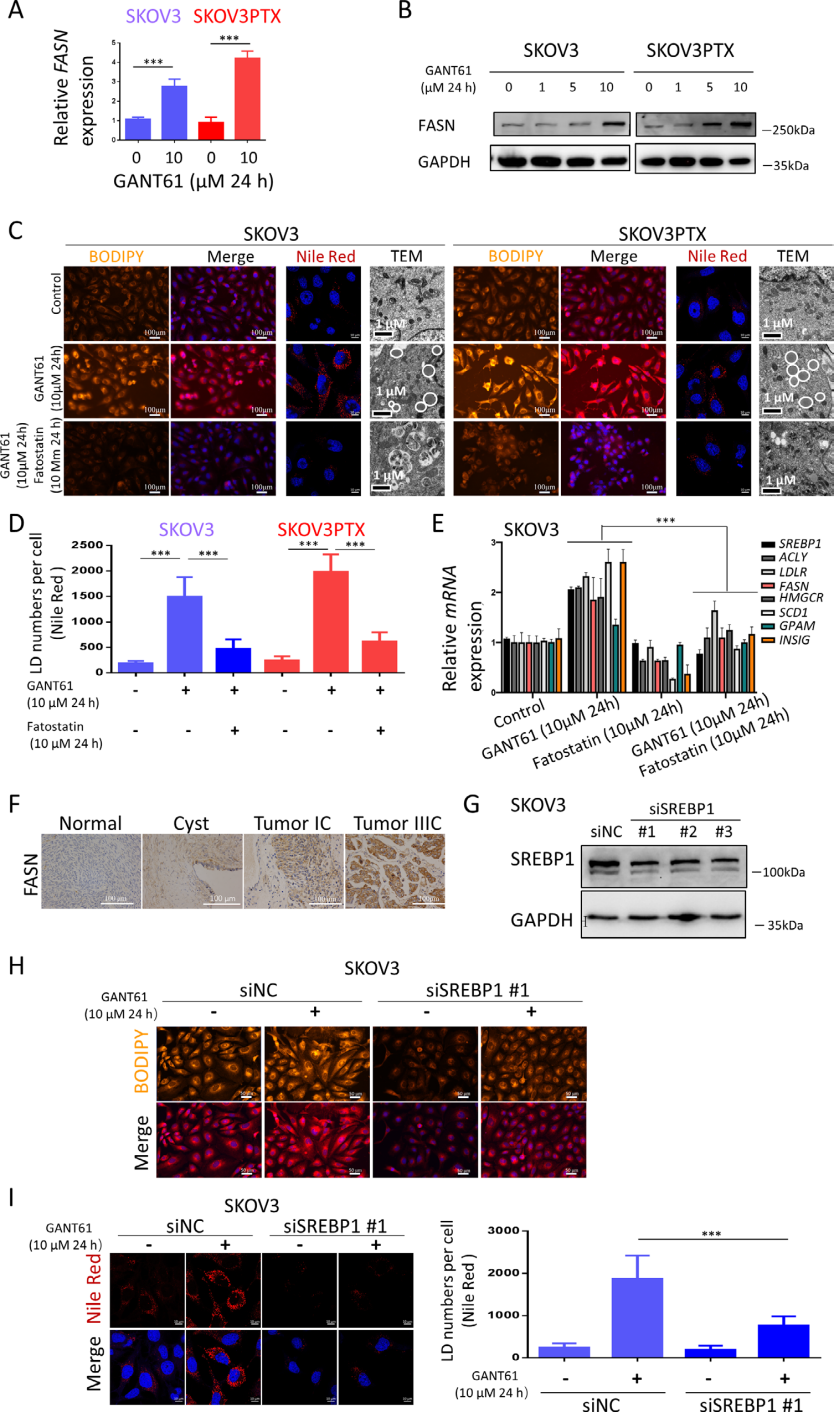
GANT61 promotes LDs accumulation in SKOV3 and SKOV3PTX cells. (A) RT‐qPCR analysis of *FASN* mRNA levels in SKOV3 and SKOV3PTX cells treated with 0 or 10 μM GANT61 for 24 h. Results represent three independent experiments, expressed as mean ± SD. (B) WB analysis of FASN protein levels in SKOV3 and SKOV3PTX cells treated with GANT61 (0, 1, 5 and 10 μM) for 24 h. GAPDH was used as a loading control. (C) Representative images of LDs by BODIPY staining, Nile Red staining and TEM in SKOV3 and SKOV3PTX cells. Cells were grouped into control, GANT61 (10 μM) and Fatostatin (10 μM) treatment for 24 h. Hoechst staining (blue) was used to label nuclei. BODIPY staining: Scale bar, 100 μm. Nile Red staining: Scale bar, 100 μm. TEM: Scale bar, 1 μm. (D) Quantification of the number of LDs by fluorescence micrscaopy (Nile Red staining). (E) RT‐qPCR analysis of mRNA levels of *SREBP1* and its downstream targets (*ACLY, LDLR, FASN, SCD‐1, HMGCR, GPAM and INSIG‐1*) in SKOV3 cells. Groups include control, GANT61 (10 μM), Fatostatin (10 μM) and GANT61 combined with Fatostatin (both at 10 μM) treated for 24 h, based on two‐way factorial ANOVA. (F) Immunohistochemical staining of FASN expression in normal ovarian tissue, ovarian serous cystadenoma, and ovarian serous carcinoma samples (*n* = 10). Scale bar, 100 μm. (G) WB analysis of SREBP1 protein expression in SKOV3 cells after knockdown of SREBP1 using siRNA (siSREBP1 #1, siSREBP1 #2, siSREBP1 #3), with GAPDH used as a loading control. (H) Immunofluorescence staining with BODIPY was used to assess changes in LDs numbers in SKOV3 cells after SREBP1 knockdown. Hoechst staining (blue) marks cell nuclei. Scale bar, 50 μm. (I) Nile Red staining was used to evaluate changes in LDs numbers in SKOV3 cells after SREBP1 gene knockdown. Quantification of the number of LDs by fluorescence micrscaopy (Nile Red staining). Scale bar, 10 μm. *p < 0.05, **p < 0.01, ***p < 0.001.

Finally, to assess the therapeutic efficacy of dual pathway inhibition, we treated SKOV3 and SKOV3PTX cells with GANT61 and Fatostatin in combination. The drug combination synergistically suppressed cellular proliferation (CCK‐8 assay) and colony‐formation capacity (Figure 5A,B). Mechanistically combined treatment induced DNA damage, as evidenced by increased γH2AX foci formation and elevated γH2AX protein expression (Figure 5C,D), while simultaneously inducing apoptosis through PARP1 cleavage (Figure 5D).

**FIGURE 5:**
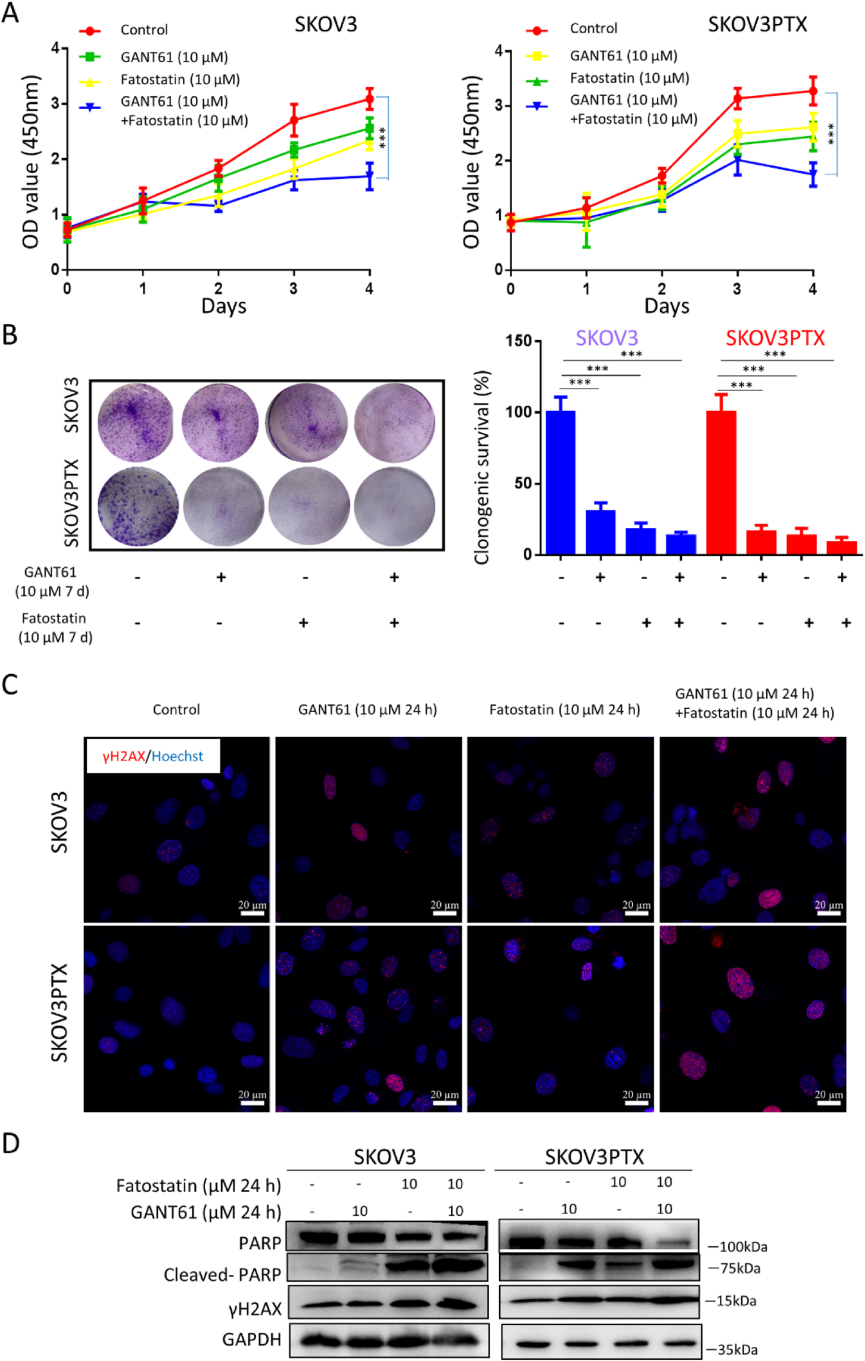
Combining GANT61 wiht Fatostatin reduces the cell viability of SKOV3 and SKOV3PTX cells. (A) CCK‐8 assay evaluating cell proliferation in SKOV3 and SKOV3PTX cells treated with the control, GANT61 (10 μM), Fatostatin (10 μM) or a combination of GANT61 (10 μM) and Fatostatin (10 μM) for 4 days. (B) Colony formation assay measuring the clonogenic potential of SKOV3 and SKOV3PTX cells after treatment with the same conditions as in (A) for 7 days. (C) Immunofluorescence staining for the DNA damage marker γH2AX (red) in SKOV3 and SKOV3PTX cells treated for 24 h with control, GANT61 (10 μM), Fatostatin (10 μM) or GANT61 (10 μM) + Fatostatin (10 μM). Hoechst (blue) was used to stain cell nuclei. Scale bar = 20 μm. (D) WB analysis of apoptotic markers Cleaved‐PARP and DNA damage protein γH2AX in SKOV3 and SKOV3PTX cells after treatment with the indicated drugs for 24 h. GANT61 (10 μM), Fatostatin (10 μM), or GANT61 (10 μM) + Fatostatin (10 μM). GAPDH was used as a loading control. *p < 0.05, **p < 0.01, ***p < 0.001.

In summary, our study highlights a multipronged therapeutic strategy for OC by combining Hedgehog pathway inhibition with autophagy or lipogenesis modulation (Figure 6). We found that SKOV3PTX cells exhibit enhanced Hedgehog signalling activation, evidenced by elevated SHH‐N and GLI1 expression compared to SKOV3 cells. Our results further indicated that GANT61 effectively inhibits the proliferation of both paclitaxel‐sensitive and resistant OC cells. Furthermore, we identified that GANT61 triggers cytoprotective autophagy, a process increasingly recognised as a therapeutic vulnerability in cancer [23, 24, 25, 26, 27]. Pharmacological autophagy inhibition with CQ synergistically enhanced the efficacy of GANT61, suggesting that GANT61‐induced autophagy plays a crucial role in its antiproliferative effects. Additionally, we uncovered a novel metabolic dimension: GANT61 activates SREBP1‐dependent lipogenesis, and its combination with the SREBP inhibitor Fatostatin showed remarkable anti‐tumour effects. These findings corroborate recent work implicating dysregulated lipid metabolism in cancer pathogenesis [28, 29, 30, 31]. Collectively, our findings position Hedgehog signalling at the intersection of autophagy regulation and metabolic reprogramming in OC. Our work provides preclinical rationale for testing GANT61‐based combination therapies, particularly in paclitaxel‐resistant cancer where current options remain limited [32, 33, 34, 35].

**FIGURE 6:**
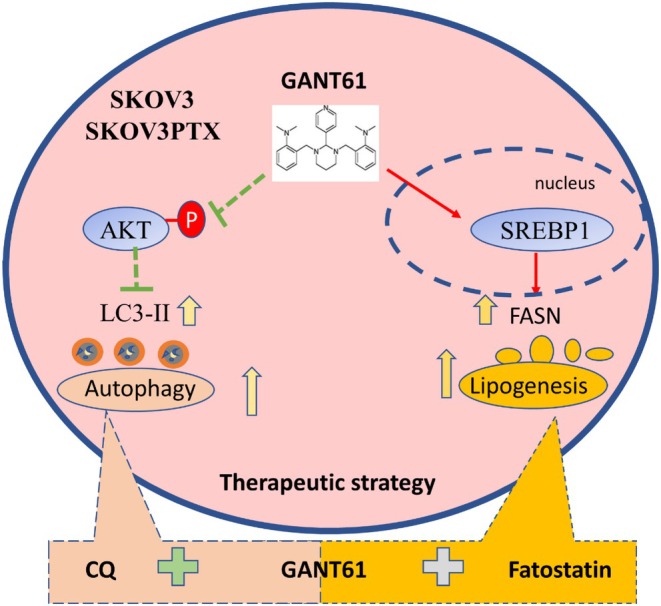
Schematic diagram of GANT61 modulates autophagy and lipid metabolism in ovarian cancer. GANT61 induced autophagy via the AKT signalling inhibition and promoted the accumulation of LDs in both cell lines. The molecular mechanism behind this lipid accumulation appears to involve the mediation of SREBP1, which promotes fatty acid synthesis in SKOV3 cells. Furthermore, combining GANT61 with CQ or Fatostatin significantly inhibited the proliferation and clonogenicity of SKOV3 and SKOV3PTX cells.

## Author Contributions

S.Z. and J.Y. provided direction and guidance throughout the preparation of this manuscript. Y.P. and L.C. wrote and edited the manuscript. Z.Z. and J.Y. reviewed and made significant revisions to the manuscript. J.S., S.H., X.G., X.M., R.T., M.L., F.S., S.S. and Y.D. collected and prepared the related papers. All authors read and approved the final manuscript.

## Ethics Statement

This work was approved by the Ethics Committee of the Sir Run Run Shaw Hospital, School of Medicine, Zhejiang University.

## Conflicts of Interest

The authors declare no conflicts of interest.

## Supporting information


**Data S1.** Supporting Information.

## Data Availability

The data that support the findings of this study are available from the corresponding author upon reasonable request.
