# Supplementary material for: GANT61 Modulates Autophagy and Lipid Metabolism in Ovarian Cancer
Source: Cell Prolif. 2025 May 1;58(7):e70051. doi: 10.1111/cpr.70051 (PMC12240631; doi:10.1111/cpr.70051)
Supplement: Supplementary file 1 — Data S1. Supporting Information. [file CPR-58-e70051-s001.docx]

**Supplementary Files**

**Supplementary Table 1. Primers used in this study**

| Primer | Sequence |
| --- | --- |
| ACLY-F | CAGAATCGGTTCAAGTATGCTC |
| ACLY-R | AAGTTTTCCACGACGTTTGATC |
| GLI1-F | GCGTGAGCCTGAATCTGTGTAT |
| GLI1-R | TGGATGTGCTCGCTGTTGATG |
| FASN-F | CCATCTACAACATCGACACCAG |
| FASN-R | CTTCCACACTATGCTCAGGTAG |
| GPAM-F | GATGTAAGCACACAAGTGAGGA |
| GPAM-R | TCCGACTCATTAGGCTTTCTTTC |
| SREBP1/2-F | CTGTGTGACCTGCTTCTTGT |
| SREBP1/2-R | CTCATGTAGGAACACCCTCC |
| SCD1-F | CTTTCTGATCATTGCCAACACA |
| SCD1-R | TGTTTCTGAAAACTTGTGGTGG |
| HMGCR-F | ATACAAGTATAGCTGGACGCAA |
| HMGCR-R | CTGCATTTCAGGGAAATACTCG |
| LDLR-F | CTGTAGGGGTCTTTACGTGTTC |
| LDLR-R | GTTTTCCTCGTCAGATTTGTCC |
| GAPDH-F | GAGGAGGCATTGCTGATGAT |
| GAPDH-R | GAAGGCTGGGGCTCATTT |

**Materials and Methods**

***Cell lines and regents***

The human ovarian adenocarcinoma cell lines SKOV3 and SKOV3PTX were obtained from the Chinese Academy of Sciences Cell Bank and the Obstetrics and Gynecology Hospital of Zhejiang University School of Medicine, respectively. Both cell lines were cultured in RPMI-1640 medium supplemented with 10% fetal bovine serum and 1% penicillin-streptomycin under standard conditions (37°C, 5% CO2).

GANT61(cat no. S2767) and Fatostatin(cat no. S9785) were purchased from Selleck Chemicals (Houston, TX, USA). Chloroquine(CQ) (cat no. C6628) was obtained from Sigma-Aldrich; Merck KGaA, Darmstadt, Germany.

Small interfering RNA (siRNA) was obtained from GuanNan.co,Ltd(hangzhou,China). Cells were transfected with 75 pmol siRNA using Lipofectamine™ 3000 Transfection Reagent(Thermo Fisher Scientific, L3000075) according to the manufacturer’s instructions. siRNA sequence for SREBP1: #1GGAGGCUUCUCUACAGGAATT; #2GCAACACAGCAACCAGAAATT; #3CCAGCAGCUCCAUUGACAATT.

***Western Blot***

Protein samples were extracted using RIPA buffer containing protease and phosphatase inhibitors, and quantified using a BCA assay. Equal amounts of protein (30–50 μg) were separated on SDS-PAGE gels and transferred onto PVDF membranes. Membranes were blocked with 5% non-fat milk in TBS-T for 1 hour at room temperature and incubated overnight at 4°C with primary antibodies diluted in blocking solution. The following primary antibodies and dilutions were used: GLI1(Abcam, ab151796, 1:1000), SHH(Abcam, Ab53281, 1:1000), GAPDH(Cell Signaling Technology, 5174, 1:5000), LC3B(Cell Signaling Technology, 43566, 1:2000) and SQSTM1/p62(Cell Signaling Technology, 23214, 1:2000), p-AKT Thr308(Cell Signaling Technology, 13038, 1:1000), p-AKT Ser473(Cell Signaling Technology, 4060, 1:1000), AKT(Cell Signaling Technology, 4691, 1:1000), S6RP(Cell Signaling Technology, 2217, 1:1000), p-S6RP(Cell Signaling Technology, 2211, 1:1000), p-mTOR(Cell Signaling Technology, 5536, 1:1000), FASN(Abcam, ab128870, 1:1000), SREBP1(Abcam, Ab3259, 1:500), PARP(Cell Signaling Technology, 9532, 1:1000), Cleaved-PARP(Cell Signaling Technology, 5625, 1:1000), γH2AX(Cell Signaling Technology, 9718, 1:1000). After washing with TBS-T (3×, 10 minutes each), membranes were incubated with HRP-conjugated secondary antibodies for 1 hour at room temperature. Signals were detected using enhanced chemiluminescence (ECL) and visualized with an imaging system. Quantification was performed using densitometric analysis software.

***RT-qPCR***

Total RNA was extracted from cells using TRIzol reagent and reverse-transcribed into cDNA using a commercial kit according to the manufacturer’s instructions. RT-qPCR was performed using a SYBR Green PCR Master Mix on a real-time PCR system. Reaction conditions included initial denaturation at 95°C for 5 minutes, followed by 40 cycles of denaturation at 95°C for 15 seconds, annealing, and extension at 60°C for 30 seconds. Relative mRNA levels were calculated using the 2^−ΔΔCt method, with GAPDH serving as the internal control. All reactions were conducted in triplicate. The information on the primers used in this study was provided in Supplementary Table 1.

***Immunohistochemistry***

Tissue sections were deparaffinized, rehydrated, and subjected to antigen retrieval using citrate buffer (pH 6.0) at 95°C for 10 minutes. After cooling, sections were blocked with 3% hydrogen peroxide and incubated with 5% serum at room temperature for 30 minutes. FASN(Abcam. ab128870, 1:200) was applied overnight at 4°C, followed by incubation with biotinylated secondary antibodies and HRP-conjugated streptavidin. Signals were developed with DAB substrate, and sections were counterstained with hematoxylin. Slides were dehydrated, mounted, and imaged under ZEISS Scope.A1.

***CCK-8 assay for drug sensitivity***

SKOV3 and SKOV3PTX cells were seeded in 96-well plates at a density of 5.0 × 10^4^ cells/mL (100 μL per well) and incubated at 37°C with 5% CO2 for 24 hours. The medium was then replaced with fresh medium containing various concentrations of GANT61, with each concentration tested in triplicate, and cells were incubated for an additional 48 hours. Subsequently, 10 μL of CCK-8 reagent was added to each well, and the plates were incubated for 2 hours. Absorbance at 450 nm was measured using a microplate reader, and values were determined using GraphPad Prism 8.0 software.

***Cell proliferation assay***

***1 CCK-8 assay for cell growth curve***

SKOV3 and SKOV3PTX cells were seeded in 96-well plates at a density of 2.0 × 10^4^ cells/mL (100 μL per well) and incubated at 37°C with 5% CO2 in triplicate. After 24 hours, cells were treated with the respective drugs, and 10 μL of CCK-8 reagent mixed with fresh medium was added to each well on days 1, 2, 3, and 4 post-treatments. Plates were incubated for 2 hours, and absorbance at 450 nm was measured using a microplate reader. OD values from days 1–4 was recorded, and cell growth curves were plotted.

***2 Colony formation assay***

Cells were seeded into 6-well plates at a density of 2000 cells per well in triplicate and incubated at 37°C with 5% CO2. Drug treatments were initiated on day 2, with media changes every other day. After 1–2 weeks of culture, colonies were fixed with 4% paraformaldehyde for 20 minutes and stained with 0.1% crystal violet for 10 minutes. Visible colonies were photographed under a microscope, and colony formation rates were quantified for statistical analysis.

***3 Real-Time Cell Analysis (RTCA)***

A RTCA system, iCELLigence (ACEA Biosciences, San Diego, CA, USA), was used to assay cell proliferation. Cells were prepared and counted, and 50 μL of culture medium was added to each well of the plate to measure the baseline. The experimental setup, including plate layout and drug concentrations, was recorded digitally. Next, 100 μL of cell suspension (10,000 cells/well) was added, and the plate was allowed to settle for 30 minutes before being placed in a real-time cell analysis system integrated with a CO2 incubator. Drug treatments were applied the following day at specified concentrations, and cells were monitored for 80 hours. Data were analyzed after completing the assay.

***Nile Red Staining***

Cells were fixed with 4% paraformaldehyde for 30 minutes, washed twice with PBS, and Nile Red (1 μg/ml) was applied for 20 minutes at 37° (protected from light), followed by 2–5 washes with distilled water. Nuclei were counterstained with Hoechst for 10 minute and washed five times with water. Images were captured using the Zeiss LSM 800 confocal laser scanning microscope.

***BODIPY staining***

Cells were washed twice with PBS, fixed with 3% paraformaldehyde for 30 minutes, and washed three times with PBS. Bodipy working solution (10 μL of 1 mg/mL BODIPY in 10 mL 150 mM NaCl) was applied for 10 minutes at room temperature, followed by nuclear counterstaining with Hoechst. Samples were rinsed once with PBS and observed under the Zeiss LSM 800 confocal laser scanning microscopea for imaging.

***Transmission Electron Microscopy***

Cells were fixed in 2.5% glutaraldehyde at 4°C for 24 hours, followed by standard protocol according to previous articles. Briefly, fixed samples were treated with 2 % osmium tetroxide for 1 hour and stained with 2 % uranyl acetate for 30 minutes, followed by dehydration, embedding, and sectioning. Ultrathin sections (80 nm) were mounted on nickel grids and imaged using an FEI Technai 12 microscope operated at 80 kV.

***Mcherry-GFP-LC3B (autophagy dual marker adenovirus) infection***

Cells were seeded and cultured to 40-50% confluence, followed by infection with mCherry-GFP-LC3B adenovirus for 48 hours. Signaling was captured using the Zeiss LSM 800 confocal laser scanning microscopea.

***Cell immunofluorescence staining***

Cells were seeded on confocal dish to 40% confluence(Saining, 1051000). After incubation for 24 h, the cells were washed twice with PBS and fixed with 4% PFA at room temperature for 10 minutes. Then, the fixed cells were further permeabilized with PBS(0.2% Triton X-100) at room temperature for 10 minutes. Primary antibodies containing LC3B(Cell Signaling Technology, 43566, 1:100), SQSTM1/p62(Cell Signaling Technology, 23214, 1:100), γH2AX(Cell Signaling Technology, 9718, 1:100), LAMP1(Abcam, ab208943, 1:100) were added to incubate at 4°C for 2 hours in a humidified compartment. After the dishes were washed three times, Alexa Fluor 594–or 488–conjugated secondary antibodies (Thermo Fisher Scientific, #A-21206; #A-11029, #A-21207; #A-21203; 1:500) were added to each well and incubated for 2 hours at 37℃. Finally, cell nucleuses were stained with DAPI for 10 min. the Zeiss LSM 800 confocal laser scanning microscopea was used to acquire the images.

***Quantitation and statistical analysis***

Data statistical analysis and graphing were conducted using GraphPad Prism 8.0 software. Each experiment was performed at least three times independently. The Shapiro-Wilk test was used to assess sample normality. For data that met the requirements of normal distribution and homogeneity of variances, differences between two groups were analyzed using unpaired t-tests. For more than two groups of normally distributed samples, one-way ANOVA was applied. A p-value < 0.05 was considered statistically significant.
